# Investigation into the fungal diversity within different regions of the gastrointestinal tract of *Panaque nigrolineatus*, a wood-eating fish

**DOI:** 10.3934/microbiol.2017.4.749

**Published:** 2017-09-04

**Authors:** Caroline L. Marden, Ryan McDonald, Harold J. Schreier, Joy E.M. Watts

**Affiliations:** 1 School of Biological Sciences, University of Portsmouth, Portsmouth, Hampshire, UK; 2 Department of Biological Sciences, University of Maryland, Baltimore County, Baltimore, MD, USA; 3 Department of Marine Biotechnology, University of Maryland Baltimore County, Baltimore, MD, USA

**Keywords:** fungi, microbiome, symbiosis, fish, *Panaque nigrolineatus*, gastrointestinal tract, wood, lignocellulose

## Abstract

The Amazonian catfish, *Panaque nigrolineatus* have several physiological adaptions enabling the scraping and consumption of wood (xylivory), facilitating a detritivorous dietary strategy. Composed of lignocellulose, wood is a difficult substrate to degrade and as yet, it is unclear whether the fish obtains any direct nutritional benefits from wood ingestion and degradation. However, there are numerous systems that rely on microbial symbioses to provide energy and other nutritional benefits for host organisms via lignocellulose decomposition. While previous studies on the microbial community of *P. nigrolineatus* have focused upon the bacterial population, the role of fungi in lignocellulose degradation in the fish has not yet been examined. This study describes the detection of fungi within the fish gastrointestinal tract. Using next generation sequencing, the effects of diet on enteric fungal populations were examined in each gastrointestinal tract region. Fungal species were found to vary in different regions of the gastrointestinal tract as a function of diet. This study is the first to examine the fungal community in a xylivorous fish and results support the hypothesis that diet influences fungal distribution and diversity within the gastrointestinal tract of *P. nigrolineatus*.

## 1. Introduction

Fungi are a diverse and ubiquitous eukaryotic kingdom that inhabit the terrestrial and aquatic environment and play a key role in global biogeochemical cycling as primary decomposers of organic material [1]. Fungal decomposition of wood is dependent on a number of factors including host species, abiotic conditions, and community composition. Three types of fungi are the predominant decomposers in the environment: white rot, brown rot, and soft rot fungi [2]. These degrade wood according to their enzymatic arsenal, breaking down cell wall polymers, penetrating the cell wall, and altering its chemistry, with the resulting constituents being taken up by the hyphae for energy and anabolism [3],[4]. Fungi and bacteria inhabit and compete for resources in ecological niches; these interactions can be powerful mutual drivers with positive and negative feedbacks [5],[6]. These bacterial-fungal interactions can be highly specific with symbiotic associations developing between bacterial cells and fungal hyphae [7].

The interactions between fungal and bacterial populations can play a critical role in the breakdown of lignocellulose in the environment [8],[9],[10]. These synergistic interactions can alter microbial community structure, development, and composition [7],[11]. Wood degradation by fungi can be inhibited or promoted by bacteria depending on the species present and the growth stage at which the association is initiated [12]. Bacteria can alter the structural integrity of wood, providing more favourable attack sites for fungi and increasing overall decomposition rates [13]. Furthermore, bacteria can also provide nutritional benefits to wood degrading fungi by supplying biologically fixed nitrogen that fungal hyphae transport to the wood degradation site [13],[14],[15].

Woody plant material is a challenging dietary resource for animals, as plants contain recalcitrant polymers including cellulose, hemicelluloses and lignins [16]. Employing a wood-based dietary strategy with its low nutritional quality and lack of nitrogenous compounds, xylivores must either rely on the activities of endosymbiotic microbes or produce the essential cellulose and lignin degrading enzymes. Some wood eating animals such as cockroaches and longicorn beetles have gut structures that imply cellulose digestion is primarily accomplished by endogenous cellulases rather than microbial cellulases [17]. In contrast, the termite gut requires a greater contribution from microbial cellulose digestion [17], which works synergistically with endogenous cellulases to enable them to degrade 74–99% of cellulose and 65–87% hemicellulose [17],[18]. A complex resident microbiota inhabits the digestive system of the bovine rumen, which converts cellulose-rich plant mass into volatile fatty acids that are subsequently absorbed by the rumen epithelium. Additional compounds such as amino acids and vitamins B and C, are also supplied to the host [19],[20].

Fish represent the greatest diversity of all vertebrates [21], however, understanding their gut microbiota and its significance is lacking compared to terrestrial vertebrates. Loricariidae is a speciose family of fish distributed in freshwater ecosystems of the Neotropics [22],[23]. One member of the Loricariidae, *Panaque nigrolineatus*, has been the focus of study by several groups due to its ability to imbibe large amounts of wood (up to 70% of the GI contents) [24]. This fish uses spoon-shaped teeth and a suckermouth to allow for ingestion of woody material by rasping [24]. Stable isotope studies provide support for the consumption of large amounts of cellulose as part of their diet [24],[25],[26], which may offer selective advantage when river nutrients are limited during the dry season [27]. The *P. nigrolineatus* gastrointestinal (GI) tract is approximately 10× its body length, providing a large surface area with many different microenvironments [24].

Previous studies have described the isolation of cellulolytic bacteria from the GI tracts and faeces of *Panaque* demonstrating the presence of a consortium of microorganisms performing cellulose breakdown [28],[29]. Using 16S rRNA and culture-based analyses, the enteric bacterial community of *P. nigrolineatus* appears distinct and specialised in each region of the GI tract. The dominant bacteria have 16S rRNA gene sequences similar to *Proteobacteria, Firmicutes, Bacteriodetes* and *Actinobacteria* [30],[31],[32]. The midgut contains phylotypes with high sequence similarity to cellulose degrading bacteria *Clostridium, Cellulomonas, Bacteroides, Eubacterium* and *Aeromonas spp.* as well as nitrogen-fixing *Bradyrhizobium* and *Agrobacterium spp.* that are capable of in situ nitrogen fixation [32]. The hindgut is dominated by *Bacteroidetes* [31],[32]. Bacterial species richness has been shown to decrease distally from foregut, through to the midgut and hindgut. While the bacterial microbiota within the GI tracts of other fish have been studied [33]–[42], comparatively little is known about the diversity, abundance, and role of the fungal microbiota in these systems [21]. It is likely that fungi play an important role in the fish microbiome, yeasts have been identified as part of the normal microbiota of fish GI tract [43]–[47] and have been studied with specific relevance for fish health and yields in aquaculture.

The GI tract of *P. nigrolineatus* is enriched with lignocellulose and provides a unique microenvironment. The fungal population within this fish warrants investigation to better understand their role in this process and possibly identify new lignocellulose degradation pathways and microbial interactions. The aim of the present study was to examine and compare the diversity of fungal communities in different GI tract regions as a function of diet. To our knowledge, this is the first description of fungal populations using molecular techniques in the GI tract of fish.

## 2. Materials and Methods

### 2.1. Fish rearing conditions and tissue preparation

*P. nigrolineatus* (L-190) were imported from South America by the fish wholesaler Aquascapesonline (Belleville, NJ). They were randomly assigned to individual, filtered, and aerated tanks kept at 29 ± 1 °C. Fish (40 mm, standard length) were fed a mixed diet of hearts of palm (*Euterpe precatoria*), algae pellets (Hikari Tropical Sinking Algae Wafers, Hayward, CA), and date palm wood (*Phoenix dactylifera*) during an acclimation period of three weeks under conditions specified by IACUC 071509JW-01. For the duration of the experiment, the fish on a mixed diet were provided with palm hearts and algae every second day while wood was constantly available. Wood was thoroughly soaked in water and autoclaved three times prior to being provided to the fish. Fish were then converted to a palm wood-only diet or a mixed diet of palm hearts and palm wood. This feeding regimen was maintained for three weeks prior to termination.

After the feeding period, one fish from each treatment was sacrificed by anaesthetic overdose in 3-aminobenzoic acid ethyl ester (MS-222, 50 mg/L) as described previously [30]. After removing the ventral body plate, sterile ice-cold phosphate buffered saline (PBS) was added to the abdominal cavity. The intestine was separated immediately distal to the stomach, removed from the body cavity, uncoiled, and measured rapidly in cold PBS. The auxiliary lobe was separated from the intestine, which was then divided into three parts of equal length, defining foregut, midgut, and hindgut regions. Tissue samples were processed using the Qiagen (Germantown, MD, USA) DNeasy Blood and Tissue Kit with pre-treatments for Gram-positive and Gram-negative bacteria according to the manufacturer's instructions. DNA extracted from three samples of each GI tract region was pooled and processed for PCR amplification.

### 2.2. Internal transcribed spacer (ITS) polymerase chain reaction (PCR) amplification and sequencing

Primers ITS1 and ITS2 were used to amplify the ITS1 region and primers fITS7 and ITS4 to amplify the ITS2 region (Table 1) using the following parameters: initial denaturation step of 2 min at 96 °C followed by 30 cycles of denaturation for 15 s at 96 °C, annealing for 30 s at 50 °C, elongation for 60 s at 72 °C. Sequencing was performed on the Illumina MiSeq V3 platform (LGC Genomics GmbH, Berlin, Germany). Barcode sequences, adapters and primer dimer products were removed from the resulting sequence fragments using Illumina bcl2fastq 1.8.4 software and submitted to GenBank (Accession numbers SRR5808488–SRR5808499).

**Table 1.** Primers used for amplification of fungal ITS rDNA genes.

Primer	Sequence (5′–3′)	Reference
ITS1	TCCGTAGGTGAACCTGCGG	[48]
ITS2	GCTGCGTTCTTCATCGATGC	[48]
ITS4	TCCTCCGCTTATTGATATGC	[48]
fITS7	GTGARTCATCGAATCTTTG	[49]

### 2.3. ITS fungal community analysis

ITS pre-processing and OTU picking was carried out with mothur 1.35.1 [50]. Sequences were subsampled in mothur to 60,000 reads per sample, with distances generated using USEARCH [51]. Chimeras were eliminated using the UCHIME algorithm [52]. The similarity threshold for ITS sequences belonging to the same operational taxonomic unit (OTU) was set to 97% and clustered by CD-HIT-EST [53] with cluster representative sequences selected based on abundance. Taxonomic classification of OTUs was performed against the UNITE v6 database [54] with species assigned at 97% identity threshold. Samples were normalised to 28,090, the lowest number of reads per sample for downstream analysis by Quantitative Insights into Microbial Ecology 1.9.0 (QIIME) [55]. Alpha diversity was measured using parallel_alpha_diversity.py script using observed_species and Chao_1_ metrics. An OTU network was generated using the make_OTU_network.py script. The resulting network was visualized in Cytoscape (3.5.1) using a spring-embedded layout.

## 3. Results

### 3.1. Distribution and diversity of fungi in the GI tract

Fungal sequences corresponding to the ITS1 and ITS2 regions were PCR amplified from all GI tract regions of both diets. A total of 256,280 sequences, clustering into 207 OTUs, were obtained from the ITS2 amplification and analysed using USEARCH. The ITS1 sequence analysis was found to be considerably less sensitive and useful for this study and the results for this region are included in supplementary information (Table S1). For the ITS2 analysis, OTUs were binned into taxonomic groupings allowing comparison of fungal community alpha diversities across tissues and diet.

**Figure 1.**
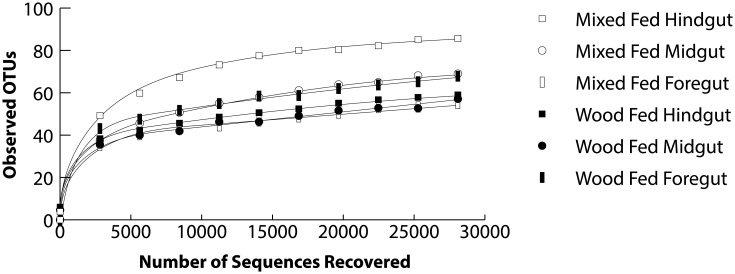
Rarefaction graphs with OTUs derived from sequencing of the ITS2 region, binned to species. The data was normalised on the sample containing the lowest number of sequences, 28,090 sequences were subsampled from the wood-fed fish and mixed-fed fish.

To detect changes in fungal alpha diversity, Chao_1_ estimations of diversity were applied to OTU distributions (Table 2). Fungal diversity increased distally with the greatest diversity in the hindgut of the mixed-diet fed fish. The opposite trend was observed in the wood-fed fish with the foregut having the most fungal diversity. These findings were supported by the rarefaction analysis, which demonstrated that the hindgut had the highest detectable species richness, while the foregut of mixed-diet fed fish had the lowest. The rarefaction analysis suggests that additional sequencing would allow more novel OTUs to be detected but the majority of the diversity present had been sampled (Figure 1). T-tests revealed there was no significant difference (*P* = 0.4) in the average number of fungal OTUs observed between wood or mixed-diet fish. Differences in S_obs_ and S_Chao1_ suggest more unique or rare OTUs being present in the wood-fed foregut (Table 2) than any other tissue region examined.

**Table 2.** Comparison of ITS2 rDNA region OTU species richness. A nonparametric estimate Chao_1_, was used to compare species diversity in different regions of the GI tract. For phylotype richness estimations, OTUs were binned to species.

	Wood Fed Fish			Mixed Fed Fish		
	Foregut	Midgut	Hindgut	Foregut	Midgut	Hindgut
Observed OTUs	67.8	57.1	59	54.9	69	85.7
Chao_1_	95.2	79.6	70	76.9	82.3	96.1

**Figure 2.**
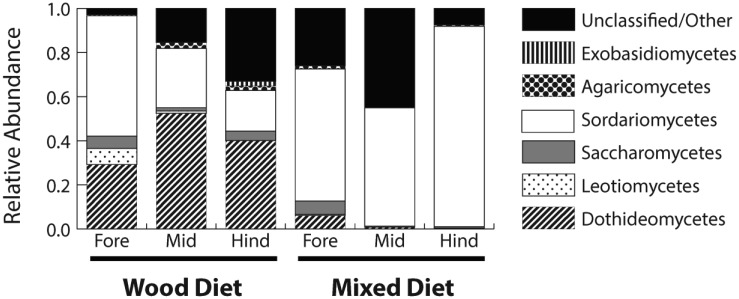
Relative abundance of dominant fungal classes (>1%) detected by sequencing the ITS2 region from the various GI tract regions of *P. nigrolineatus* fed either a wood diet or a mixed diet. Sequences were assigned to OTUs with over 97% sequence identity.

**Figure 3.**
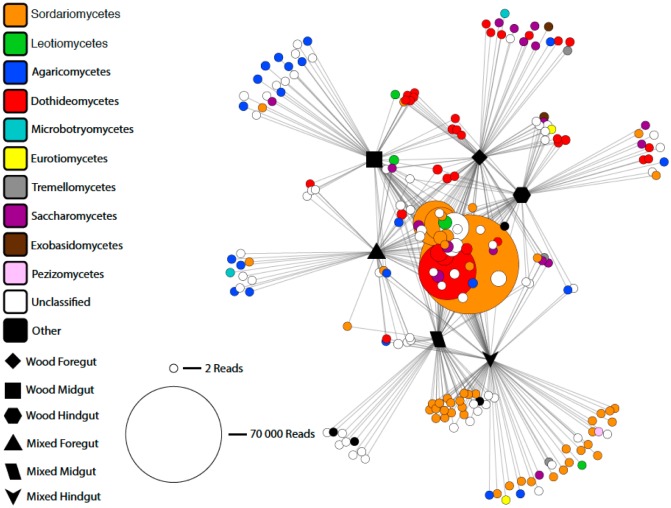
OTU network showing distribution of all OTUs identified to class detected via sequencing of the ITS2 region from different regions of the GI tract of *P. nigrolineatus* fed either a wood diet or a mixed diet. Node size indicates the number of reads assigned to an OTU while node colour indicates consensus taxonomy.

The taxonomic composition of the microbial community varied across tissue type and diet (Figure 2, Figure 3 and Figure S1). Figure 3 provides visualisation of fungal OTUs unique to each fish GI tract region and those that were shared between two or more regions. Different OTUs were detected within each region of each fish, but the core microbiome of the fish is dominated by sequences with high similarity to Sordariomycetes and Dothideomycetes in all regions with the former more prevalent in the midgut and hindgut of the mixed-diet fed fish and the latter dominated in the midgut and hindgut of the wood-diet fed fish (Figure 3). Sequences with high sequence similarity to *Fusarium oxysporum* were the major OTUs detected in all tissue regions, except the wood-fed hindgut, which was dominated by sequences similar to *Cirrenalia macrocephala.* Other sequences present throughout the GI tracts of both dietary regimens included *Aureobasidium pullulans* and *Debaryomyces prosopidis* (both more abundant in wood-fed fish), and *Malassezia restricta*.

### 3.2. Relative distribution and diversity of fungi based on dietary regimen

OTUs were binned into taxonomic groupings allowing comparison of fungal diversity within the GI tract of wood-fed fish and mixed-fed fish, irrespective of GI tract region. The GI tract of the mixed-fed fish was found to have the most ITS2 region sequence diversity. Chao_1_ analysis indicated that the wood-fed fish had a higher number of rare and unique species compared to mixed-diet fed fish (Table 2). The most prevalent classes in the wood-diet fed fish were sequences similar to Dothideomycetes (40%) and Sordariomycetes (36%) and the mixed-diet fed fish were sequences similar to Sordariomycetes (73%). Three OTUs detected from the Dothideomycetes class were found in the foregut, midgut and hindgut of the wood-diet fed fish but absent in the mixed-diet fed fish.

Each region of the GI tract was analysed for differences in community composition. Analysis did not reveal any tissue specific fungal communities, but sequences similar to the Saccharomycete genus *Metschnikowia* were found exclusively in the foregut of both fish while OTUs with sequences similar to Tremellomycetes and the Agaricomycete genus *Stereaceae* were found solely in the hindgut. These OTUs were not found in high abundance in either feeding regimen suggesting limited biological significance. However, analyses were based on the sequences of one foregut, midgut and hindgut from each diet and may not reflect actual variability amongst individuals [57].

## 4. Conclusions

In this study, the fungal communities of wood and mixed-diet fed *P. nigrolineatus* GI tracts were investigated via rDNA ITS sequencing. Due to complications associated with fish acquisition and rearing, we were only able to analyse one fish raised on each diet. While we recognise that a rigorous analysis of the GI tract communities requires the utilisation of several fish, we found that each GI tract region possessed a distinct fungal community and this is the first report of the presence of fungi throughout a fish GI tract.

*P. nigrolineatus* imbibes large quantities of wood in its diet and may have developed a symbiotic relationship with microbes to degrade this resource. Wood decomposition is dynamic and the rate of decomposition depends on many factors including priority effects [58] and successional changes in microbial communities [59]. Fungi have been shown to shape the composition of the bacterial communities [60] and are thought to be more abundant in the early stages of decay, with fungal mycelia doubling faster than bacterial cells [61], and bacterial-fungal interactions facilitating decomposition [7],[11],[60]. Although bacterial-fungal interactions are vital in plant cell wall digestion, aerobic and anaerobic fungal activity has been shown to be responsible for most cell wall degradation by penetrating into plant tissues not normally available to bacteria [62].

Our finding that *P. nigrolineatus* fed a wood-diet or a mixed-diet has different fungal communities is consistent with previous and current research showing similar variations for the bacterial communities in different regions of the GI tract [30, McDonald, Watts and Schreier, manuscript in preparation]. Of particular interest is the hindgut, which contains cellulolytic bacteria [29],[30],[31] as well as sequences similar to *C. macrocephala*, which has been associated with waterlogged wood [63],[64]; any relationship between these bacteria and fungi and their role in the fish GI tract remains to be determined.

Combined with previous studies [29]–[32], our results indicate that bacteria and fungi co-inhabit the GI tract of *P. nigrolineatus*, with the potential of degrading dietary wood. Ongoing studies [30, McDonald, Watts and Schreier, manuscript in preparation] suggest that the enteric bacterial community may lack selected enzymatic activities essential for lignocellulolytic digestion, which may be provided by the fungal community and/or host. ITS1/ITS2 sequences for several cellulase-producing fungi have been found in the present study, including those having similarity with *F. oxysporum*, *A. pullulans, Botrytis caroliniana, Metschnikowia*, *Alternaria* and *Debaryomyces*. *F. oxysporum*, which dominated foregut and midgut regions, excretes enodocellulases, exocellulases and β-glucosidase [65]. These cellulolytic activities might allow the bacteria to benefit from the primary stage of cellulose degradation as part of a synergistic relationship. While there are different fungi in different regions of each fish, many may be carrying out equivalent roles, acting on cellulose to enhance and augment bacterial activities. Future studies will examine whether genes utilised for wood degradation are differentially expressed.

While we have identified the fungal microbiota within the GI tract, the type of symbiotic relationship that this partnership takes with bacteria and host is unknown. It is conceivable that during the breakdown of lignocellulose, microbes produce volatile fatty acids and amino acids, which are absorbed by the fish and provide a source of energy. Bacteria facilitate fungal decomposition of lignin [5],[7] by altering wood chemistry, structure, and permeability [13]. They may also provide nutritional benefits to wood degrading fungi via nitrogen fixation, allowing fungi to decompose nitrogen-sparse wood [15]. An active nitrogen-fixing community has been identified in the GI tract of *P. nigrolioneatus* [32], suggesting that a mutualistic symbiosis between fungi and bacteria is possible within the fish GI tract. Confirmation of such a relationship will require further studies.

Fungi are commercially important and play a critical role in global environmental health. There is considerable interest in applied fungal research, which includes commercial production of important compounds, human health, food safety and security and crop protection [66]. Most research to date has focused on fungi that affect human health or have commercial applications. Comparatively little is known about many of the fungal species found in the environment. Analysing fungal community roles and identities in a novel environment is challenging due to the paucity of literature and lack of available sequences. The quality of fungal sequence databases is highly variable [67] and often lacks proper annotation and lineage designations [66],[68]. Amplifying the entire ITS region (including the 5.8S region) may be more phylogenetically informative, but may also artificially reduce microbial richness and bias community structure [69]. In addition, the incidence of chimeric sequences also increases due to the conservation of the 5.8S region [70]. In this study, both ITS1 and ITS2 were used for community analysis. Although, both sets of primers used for their amplification are biased for certain groups [49],[71]. In this study, the ITS2 region was found to be more sensitive in detecting novel OTUs, which could be due to the sample type or the biases and limitations of the primer sets [49],[67],[70].

Different fungal communities were detected across tissue regions and dietary regimens, indicating diet and tissue type affects fungal diversity in fish. Since *P. nigrolineatus* does not appear to gain energy directly from the digestion of wood [26],[72] it is possible that enteric fungal communities are important for wood-only diets that lacks readily available carbon and nitrogen by supplying a digestible source of carbon or other micronutrients. This study is the first to examine the fungal community in a xylivorous fish and our results indicate the presence of a diverse fungal population that may play critical roles in cellulose degradation with potential nutritional benefits for the fish. Furthermore, these previously under-studied fungal species may have novel cellulolytic and lignin degrading capabilities that could have implications for biofuel generation. Understanding the role of the fungal communities in lignocellulose degradation and their interaction with GI tract bacteria in this process is the focus of future studies.
