# Supplementary material for: Investigation into the fungal diversity within different regions of the gastrointestinal tract of Panaque nigrolineatus, a wood-eating fish
Source: AIMS Microbiol. 2017 Sep 4;3(4):749–61. doi: 10.3934/microbiol.2017.4.749 (PMC5687512; doi:10.3934/microbiol.2017.4.749)
Supplement: Supplementary file 1 [file microbiol-03-04-749-s001.pdf]

AIMS Microbiology

AIMS Microbiology, 3(4): 749-761.
DOI: 10.3934/microbiol.2017.4.749
Received: 27 June 2017
Accepted: 25 August 2017
Published: 04 September 2017

http://www.aimspress.com/journal/microbiology

Research article

Investigation into the fungal diversity within different regions of the gastrointestinal tract of *Panaque nigrolineatus*, a wood-eating fish

Caroline L. Marden [1], Ryan McDonald [2], Harold J. Schreier [2,3], and Joy E.M. Watts [1,*]

[1] School of Biological Sciences, University of Portsmouth, Portsmouth, Hampshire, UK
[2] Department of Biological Sciences, University of Maryland, Baltimore County, Baltimore, MD, USA
[3] Department of Marine Biotechnology, University of Maryland Baltimore County, Baltimore, MD, USA

* **Correspondence:** Email: joy.watts@port.ac.uk; Tel: +44-(0)2392846211;
Fax: +44-(0)2392842070.

Supplementary Information

Table S1. Total number of phyla OTU clusters from sequencing the fungal ITS1 and ITS2 regions. Unknown sequences could not be identified as fungi.

Consensus lineage	Total OTUs	
	ITS1	ITS2
Ascomycota	75	117
Basidiomycota	30	35
Blastocladiomycota	1	0
Glomeromycota	1	0
Zygomycota	1	0
Protista	0	4
Unknown fungi	57	32
Unknown sequences	25	19

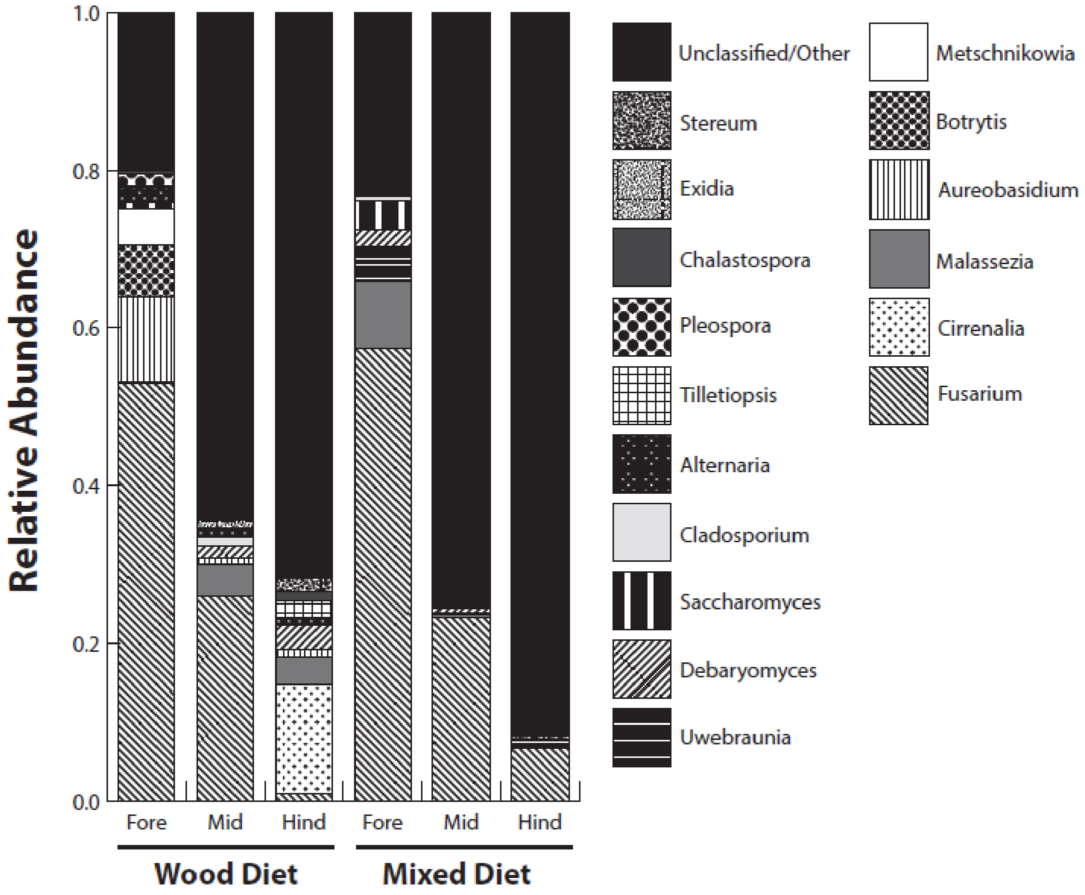

Figure S1. Relative abundance of dominant fungal genera (>1%) detected by sequencing the ITS2 region from different GI tract regions of *P. nigrolineatus*, fed either a wood diet or a mixed diet. Sequences were assigned with 97% sequence identity.

Distribution and diversity of fungi in the GI tract

Fungal sequences corresponding to the ITS1 region was PCR amplified and samples were sequenced and analysed using USEARCH. A total 290,524 sequences clustered into 190 OTUs, which were binned into taxonomic groupings allowing comparison of fungal community alpha diversity of different enteric tissues of fish fed a different diet.

To detect changes in fungal alpha diversity, $Chao_1$ estimates were calculated (Table S2). Fungal diversity was highest in the foregut of the mixed-diet fed fish, decreasing through the midgut to the hindgut. The wood-diet fed fish shows similar results in all regions with the midgut having higher fungal diversity than the fore and hindgut. These findings were confirmed by rarefaction analysis, which demonstrated that mixed-diet fed foregut had the highest detectable species richness and mixed-diet fed hindgut had the lowest. A t-test revealed there was no difference (P = 0.29) in the average number of fungal OTUs observed between wood or mixed-diet fish. Differences in S_{obs} and S_{Chao1} suggest many unique or rare OTUs were found (Table S2).

Table S2. Statistical analysis of ITS1 rDNA region OTU species richness. A nonparametric estimate $Chao_1$ was used to compare species diversity in different regions of the GI tract. For phylotype richness estimations, OTUs were binned to species.

	Wood Fed Fish			Mixed Fed Fish		
	Foregut	Midgut	Hindgut	Foregut	Midgut	Hindgut
Observed OTUs	58	59.5	52	84.6	74.6	52.4
$Chao_1$	59.2	63.7	52.3	94.2	93.1	78.1

Using a relative comparison of OTU abundances, the composition of the microbial community changed per tissue type and diet. Many more sequences were either unknown, unknown fungi or identified to phylum level compared to sequencing of the ITS2 region. There were sequences similar to only two species found in all enteric regions of both fish, Sordariomycetes represented by *Fusarium* and *Scedosporium*, with the former decreasing distally in both fish and the latter being more dominant in the hindgut of both fish.

AIMS Press

© 2017 Joy E.M. Watts, et al., licensee AIMS Press. This is an open access article distributed under the terms of the Creative Commons Attribution License (http://creativecommons.org/licenses/by/4.0)
